# CONTACT: a non-randomised feasibility study of bluetooth-enabled wearables for contact tracing in UK care homes during the COVID-19 pandemic

**DOI:** 10.1186/s40814-024-01549-6

**Published:** 2024-10-02

**Authors:** Carl A. Thompson, Thomas Willis, Amanda Farrin, Adam Gordon, Amrit Dafu-O’Reilly, Catherine Noakes, Kishwer Khaliq, Andrew Kemp, Tom Hall, Chris Bojke, Karen Spilsbury

**Affiliations:** 1 School of Healthcare, University of Leeds, Baines Wing, Leeds, LS2 9JT UK; 2 Leeds Institute of Clinical Trials Research, University of Leeds, Leeds, LS2 9JT UK; 3 Academic Centre for Healthy Ageing, Queen Mary University, London, E1 2AD UK; 4 School of Civil Engineering, University of Leeds, Leeds, LS2 9JT UK; 5 School of Electronics and Electrical Engineering, University of Leeds, LS2 9JT Leeds, UK; 6 South Tyneside Council, South Shields, NE33 2RL UK; 7 School of Medicine, Academic Unit of Health Economics, University of Leeds, Leeds, LS2 9JT UK

**Keywords:** Digital contact tracing, Care homes, Bluetooth-enabled wearables, Long-term care, Feasibility, COVID-19, Complex interventions

## Abstract

**Background:**

The need for effective non-pharmaceutical infection prevention measures such as contact tracing in pandemics remains in care homes, but traditional approaches to contact tracing are not feasible in care homes. The CONTACT intervention introduces Bluetooth-enabled wearable devices (BLE wearables) as a potential solution for automated contact tracing. Using structured reports and reports triggered by positive COVID-19 cases in homes, we fed contact patterns and trends back to homes to support better-informed infection prevention decisions and reduce blanket application of restrictive measures. This paper reports on the evaluation of feasibility and acceptability of the intervention prior to a planned definitive cluster randomised trial of the CONTACT BLE wearable intervention.

**Methods:**

CONTACT was a non-randomised mixed-method feasibility study over 2 months in four English care homes. Recruitment was via care home research networks, with individual consent. Data collection methods included routine data from the devices, case report forms, qualitative interviews (with staff and residents), field observation of care, and an adapted version of the NoMaD survey instrument to explore implementation using Normalisation Process Theory. Quantitative data were analysed using descriptive statistical methods. Qualitative data were thematically analysed using a framework approach and Normalisation Process Theory. Intervention and study delivery were evaluated against predefined progression criteria.

**Results:**

Of 156 eligible residents, 105 agreed to wear a device, with 102 (97%) starting the intervention. Of 225 eligible staff, 82% (*n* = 178) participated. Device loss and damage were significant: 11% of resident devices were lost or damaged, ~ 50% were replaced. Staff lost fewer devices, just 6%, but less than 10% were replaced. Fob wearables needed more battery changes than card-type devices (15% vs. 0%). Structured and reactive feedback was variably understood by homes but unlikely to be acted on. Researcher support for interpreting reports was valued. Homes found information useful when it confirmed rather than challenged preconceived contact patterns. Staff privacy concerns were a barrier to adoption. Study procedures added to existing work, making participation burdensome. Study participation benefits did not outweigh perceived burden and were amplified by the pandemic context. CONTACT did not meet its quantitative or qualitative progression criteria.

**Conclusion:**

CONTACT found a large-scale definitive trial of BLE wearables for contact tracing and feedback-informed IPC in care homes unfeasible and unacceptable — at least in the context of shifting COVID-19 pandemic demands. Future research should co-design interventions and studies with care homes, focusing on successful intervention implementation as well as technical effectiveness.

**Trial registration:**

ISRCTN registration: 11204126 registered 17/02/2021.

**Supplementary Information:**

The online version contains supplementary material available at 10.1186/s40814-024-01549-6.

## Key messages regarding feasibility

- What uncertainties existed regarding the feasibility?

No one has undertaken a randomised clinical trial of Bluetooth-enabled (BLE) wearables for contact tracing, a key non-pharmaceutical infection protection and control intervention in long-term care homes. Wearables have shown promise in simulation and modelling studies, but optimal implementation strategies and study procedures for homes participating in a cluster randomised trial are uncertain and untested.

- What are the key feasibility findings?

Despite technical efficacy of the technology, real-world use of BLE wearables in a care home environment and standard procedures in a cluster randomised trial context was not feasible or acceptable to homes — at least not in a pandemic context.

- What are the implications of the feasibility findings for the design of the main study?

The care home context, amplified by pandemic conditions and demands, means any cluster randomised trial of BLE wearables needs to spend sufficient time on co-designing a theory and evidence-informed implementation strategy for any intervention as well as robust designs for evaluating effectiveness if it is to be acceptable and feasible to homes. 

## Background

COVID-19 disproportionately harmed residents and staff of long-term care homes (nursing and residential homes). In England and Wales, almost 17% of the 274,063 deaths in care homes between March 2020 and February 2022 were COVID-19 related, with the virus implicated in 14% of 9175 deaths of social care staff [1]. Globally, COVID-19 accounted for 429,265 care home resident deaths between February 2020 and April 2022 [2]. The highly transmissible, airborne nature of SARS-CoV-2 in confined spaces, and widespread frailty amongst residents, rendered care homes particularly vulnerable [3].

Vaccines have not eradicated COVID-19. Non-pharmaceutical infection prevention and control (IPC) measures such as entry regulation (lockdown and quarantine), contact regulation (contact tracing, physical distancing, isolation), transmission reduction measures (screens, masks, surface cleaning), and surveillance (regular testing) will remain important for homes in any future pandemics [4]. IPC measures are often applied on a blanket basis to whole homes, despite needing more high-quality research to validate assumed effectiveness [4].

Contact tracing disrupts infection transmission by identifying and managing individuals exposed to infected people. Its effectiveness hinges on speed, timing, and population tracing comprehensiveness [5, 6]. In addition to COVID-19, contact tracing can mitigate infections and deaths from communicable diseases like influenza, norovirus, salmonella, and *Streptococcus pyogenes*, respiratory syncytial virus (RSV) which account for over 50% of care home infections [7].

Traditional contact tracing involves recalling and stating recent contacts, analysing documentary or observational evidence, or using smartphone Bluetooth or GPS capabilities. These are unrealistic methods for care homes as dementia and memory problems impact 70–80% of residents [8], documentation is sometimes of questionable validity as a record of care delivered [9], smartphone use by residents is far less than the ~ 60% population coverage required for effective tracing [10], and staff may be discouraged from using phones at work.

**Fig. 1:**
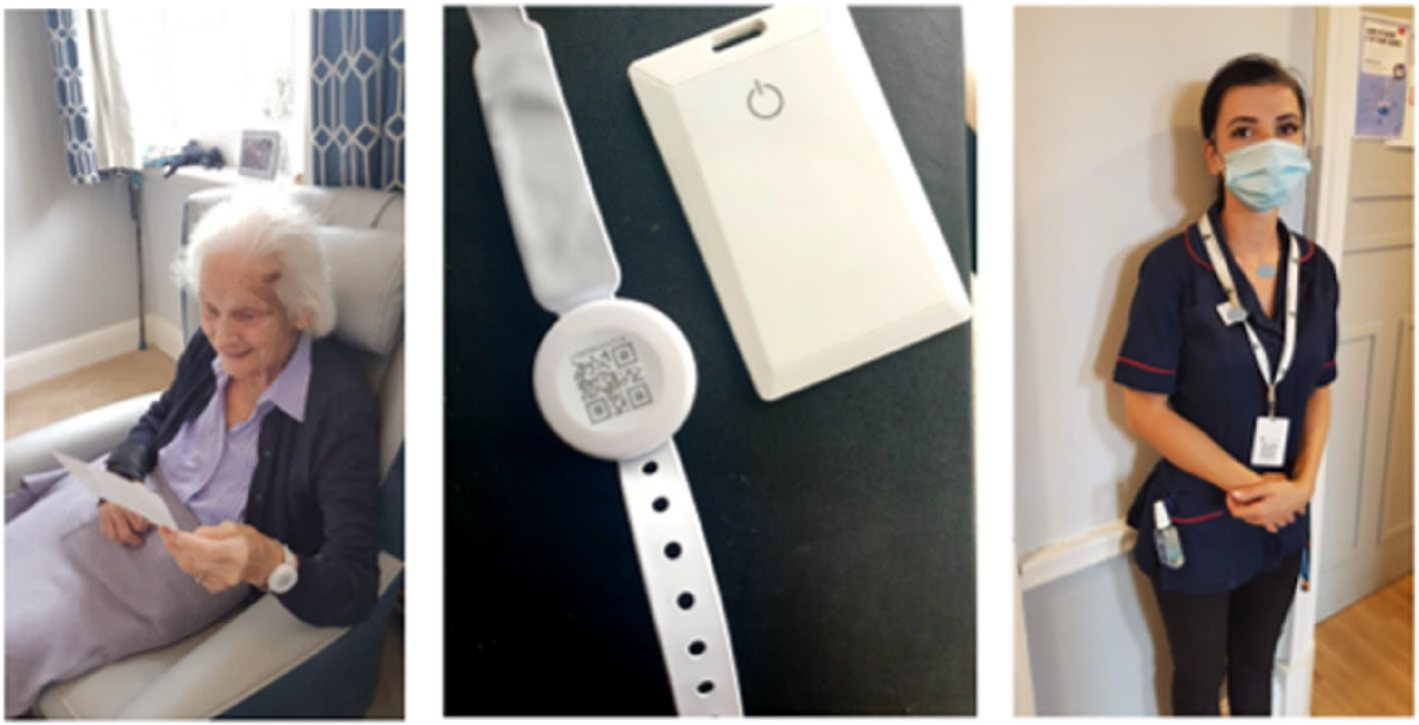
BLE wearable forms in a care home

An alternative is systems built around Bluetooth-enabled wearable devices (BLE wearables). BLE wearables harness Bluetooth, low-frequency wide-area networks/LoRaWAN, and the Internet of Things (IoT) to collect and transmit data on contacts between wearables and IoT devices (who, when, duration, proximity, and location). Wearables can be deployed as fobs, wristwatches, brooches, or cards on lanyards (see Fig. 1). In settings other than care homes, BLE wearables have shown promise for analysing proximity networks in healthcare [11] and simulation-based modelling of adoption and infections [12]. Given the pandemic context and urgency (the study was commissioned pre-vaccine development and roll-out) of the need to improve the effectiveness of isolation whilst minimising the harms associated with isolation for older people, wearables provided a potentially rapid, automated, and scalable solution for contact tracing in care homes.

### The CONTACT intervention

The CONTACT intervention was a BLE wearable and IoT system for collecting data on contacts and feeding back information contact patterns and trends to care homes. BLE wearables and location markers were deployed to pinpoint contact locations. In collaboration with the PROTECT COVID-19 National Core Study team, we also installed air quality sensors (in two homes) to monitor CO_2_ levels, temperature, and humidity [13].

Sensor placement was based on home-supplied floor plans, focusing on areas with high foot traffic, such as communal lounges and dining rooms, and extending to selected bedrooms, staff areas, and key infrastructure (e.g. kitchens). CONTACT’s components had a unique QR code identifier allowing us to map each home’s system, which took two researchers approximately four hours to install.

Consenting staff and residents wore a device whilst in the home. Each device and location marker’s unique identifier enabled secure de-anonymisation for contact and location tracing purposes by the homes.

Contact events (data) from the wearables and location markers were transmitted via a “wave” [14] scanner to a Long-Range Wide-Area Network (LoRaWAN) gateway and our commercial partner’s (MicroShare®) network. Anonymised data on devices, location marker IDs, and timestamps were sent to our Clinical Trials Research Unit for analysis: summaries of contacts, trends, and infection risks. These provided the basis of feedback to the homes (see Appendices A and B).

Feedback was delivered in a structured monthly report (see Appendix A), with ad hoc reports, triggered by notification of COVID-19 positive cases (Appendix B) detailing contacts between infected residents and other users. Information was presented back to homes at individual and aggregate levels: who had contact with whom, when, where and duration of contact, and mean numbers of contacts, aggregate COVID-19 risk, and where most contacts happened. The monthly feedback reports were delivered on the 1st of each calendar month and ad hoc/*triggered* reports within 24 h of notification of cases from homes. We did not specify how, or how quickly, homes should “act” on reports. Exploring whether, and how, actions arose was part of the acceptability of the intervention.

Reports were based on principles of effective feedback [15], and co-designed with homes' “study champions”. These were one or sometimes two individuals appointed by homes to take the lead on CONTACT study tasks, advocating for the study and acting as a point of contact between homes and researchers. Evolutionary changes based on staff feedback included adding key messages from the research team and simplifying the visual representation of infection trends. Reports were emailed to the homes. A researcher followed up 3 days later to address any questions, with interactions documented for our embedded process evaluation [16].

### Rationale

BLE wearables make contact tracing in homes feasible by collecting and transmitting significant contact data, filling an information deficit for homes [17, 18]. Providing accurate contact information to those in charge of a care home’s IPC could lead to better-informed, higher-quality, decisions, potentially reducing infections and avoiding blanket application of often restrictive non-pharmaceutical interventions (“lockdowns”) to individuals regardless of their infection risk.

### Research aims

CONTACT aimed to determine the feasibility and acceptability of a BLE wearable-based contact tracing system amongst care home residents and staff.

We had three main objectives informing our decision to proceed to a definitive cluster randomised controlled trial of CONTACT versus infection prevention and control as usual in homes:

i) Assess the acceptability and feasibility of intervention delivery processes, by evaluating
  - The *contact tracing devices* and *wider system*
  - The *tailored feedback*
  - *Intervention delivery* and *site engagement*
ii) Assess the acceptability and feasibility of study design/implementation processes, by evaluating
  - *System software*
  - *Main study delivery potential*
  - *Data collection*
iii) Decide to progress (or not) to main trial by
  - *Evaluating progress against predefined criteria*

## Methods

CONTACT was a non-randomised mixed-methods feasibility study [19] with an embedded parallel process evaluation [16]. It followed a protocol available at https://njl-admin.nihr.ac.uk/document/download/2035361, with ethical approval from the UK Health Research Authority (REC: 294,390). A key change from protocol was an initially planned web-based “dashboard” for real-time, continuously updated reports for each home that was dropped due to a lack of demand from homes.

### Participants

#### Eligibility

CONTACT was a whole-home intervention. We included all residents, staff, and visitors willing to wear a device, barring exceptions such as residents with disorders like pica that could pose a risk. Eligible homes needed to assign a champion, promote the study, free staff for training, implement the intervention, provide data, and participate in the process evaluation.

### Identification and consent

Homes were recruited using care home research networks (National Institute for Health Research ENRICH [20]; NICHE-Leeds [21]). Selection was based on location, staffing, registration type, and resident characteristics (see Table 1). Whole-home consent was initially planned, but managers’ (in two of the homes) perceptions of wider regulatory requirements meant individual consent processes were used. Individual consent was sought from residents, staff, and nominees/consultees for incapacitated residents.

**Table 1:** CONTACT feasibility study care homes

**Home**	**Type**^a^	**Ownership** [22]	**Maximum capacity**	**Number of staff**	**Number of residents**	**Number of residents with dementia**	**Device type issued**
Home 1	Residential care	For-profit independent	30	25	26	6	Card
Home 2	Residential care	For-profit independent	15	21	15	2	Card
Home 3	Nursing care	For-profit independent	28	37	23	5	Fob
Home 4	Dual registered for residential and nursing care	For-profit non-private equity chain	102	120	87	25	Fob

^a^Residential care homes offer a safe environment for support with personal care, like dressing and washing, activities, and opportunities for socialising. Alongside opportunities for socialising, nursing homes provide registered nursing care for those with higher levels of care need (for example, post hospital discharge or with long-term care needs arising from conditions such as dementias). Nursing homes have a qualified nurse on site round-the-clock, supported by care assistants, so they can provide a higher level of care

### Data collection settings and location

Data were collected in four care homes (Table 1).

Home One, in urban West Yorkshire, was a for-profit residential home run by an employed manager. It had a staff:resident ratio of 1:1 and was a converted large house, with experience in previous research studies.

Home Two, a small, owner-managed, for-profit residential care home in rural West Yorkshire, had a staff:resident ratio of 1.4:1. It was purpose-built and had limited research experience.

Home Three was an owner-managed for-profit home in affluent North Yorkshire. It was housed in a converted Victorian property, with a staff:resident ratio of 1.6:1. Around 25% of the residents lived with dementia.

Home Four was a family-run, non-private equity-owned home with both nursing and residential care provisions. It was in a converted factory with large communal areas. It had a staff:resident ratio of 1.4:1. Three floors catered to residents with differing needs (residential, nursing, and dementia).

All homes were rated good by the Care Quality Commission at point of recruitment and demonstrably committed to the study.

### Data and analysis

Data were verified against a participant list and checked for an appropriate inter-device signal strength. Data not meeting these conditions were excluded.

Physical distance between CONTACT wearables was calculated thus:

$$\mathrm{Distance}=10^{\,\wedge}\left((\mathrm{Measured}\;\mathrm{Power}-\mathrm{RSSI})/\;(10\ast\mathrm N)\right)$$

RSSI (Received Signal Strength Index) was the signal strength as measured by the receiving device. A signal strength of ≤ 75 equated to ≤ 2 m. Time was measured in seconds. Contact between devices was in line with government guidance on clinically significant contacts at the time of the study [23].

We assessed home adherence to study procedures and device management qualitatively, examining study fault logs, weekly support call notes, and process evaluation interviews and observations. This approach accompanied our formal feasibility evaluation against progression criteria.

Home managers completed an adapted version of the NoMaD questionnaire [24] to assess perceptions of factors relevant to enabling CONTACT as routine work. NoMaD has good face validity, construct validity, and internal consistency [24].

Quantitative data (including time) was collated, cleaned, and described using summary measures of central tendency, variability, missing values, and bias. Qualitative data were analysed using a framework analytic approach and NPT-informed coding matrices and guidance for developing and evaluating complex interventions [25]. A more detailed explanation of our qualitative analysis is reported in CONTACT’s process evaluation [16].

### Outcomes

Table 2 outlines the data collection associated with outcomes and study objectives.

**Table 2:**
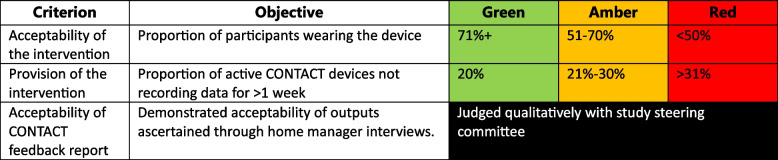
CONTACT progression criteria

#### Prespecified progression criteria

We evaluated the acceptability and implementation of the CONTACT intervention after 2 months at the study end (see Table 3).

**Table 3:** Baseline characteristics of staff and residents

	**Residents (*n* = 102)**	**Staff (*n* = 158)**
Mean age (SD)	86.1 (8.58)	42.1 (14.75)
Male	27 (27%)	20 (12%)
Female	73 (73%)	137 (87%)
Ethnicity: White	101 (100.00%)	
Length of time in care home in weeks, median (range)	99.5 (2, 590)	
Previous + COVID-19 test	20 (20%)	41 (26%)
Weeks since + test, median (range)	47.0 (22, 65)	46.0 (3, 88)
COVID-19 vaccinated	99 (99%)	154 (99%)
Dementia diagnosis	38 (38%)	
Dementia severity: mild	9 (24%)	
Dementia severity: moderate	18 (49%)	
Dementia severity: severe	10 (27.03%)	
Length of employment in home in weeks, median (range)		123.5 (0, 1302)
Employment status		
Permanent		140 (90%)
Bank		15 (10%)
Role		
Direct care/nursing staff		101 (64%)
Specialist non-clinical role		1 (1%)
Manager		6 (4%)
Estates/maintenance		3 (2%)
Clerical/administrative		7 (4%)
Catering		17 (11%)
Cleaner		11 (7%)
Other (please specify)		11 (7%)
Work in more than one home: yes		1 (1%)
Work in more than one home: no		157 (99%)

Analysis for progression to main trial was based on descriptive statistics (percentages) rather than formal hypothesis testing. Baseline characteristic summaries of each care home and participants were produced and numbers (percentages) were calculated for binary and categorical outcome measures, whilst mean (variance) was summarised for any continuous outcomes [26]. Feedback report acceptability was a research team judgement based on qualitative analysis of interviews with home managers.

### Sample size rationale

CONTACT was an entirely novel intervention, in an unprecedented pandemic context with a diverse (and challenging to undertake trials with) population of participants; uncertainty and variability were high. The absence of data on care home residents and staff use of BLE wearables meant pre-study, evidence-based, estimation of parameters and confidence intervals was not possible. We focused instead on generating preliminary data to inform estimates for a main trial, and ensuring estimates would be based on trustworthy, context-specific, high-quality data: reducing the risk of over/underestimating key parameters and compromising any future trial. Accordingly, and in line with feasibility study guidelines [27], no formal power calculation was undertaken. Appropriate rules of thumb for feasibility study sample sizes range from 24 to 50 participants [28–30]. With our four homes each comprising between 36 and 207 participants (see Table 1), we were satisfied that we would have enough participants to allow for estimates of confidence intervals for relevant parameters (see Tables 2 and 6) in any future trial.

For the qualitative study component, we purposively (on theoretical and pragmatic grounds) selected staff based on qualifications (including registered nurses and non-registered care staff), their responsibilities (including team leaders and those in managerial roles), and roles (including care and non-care roles like administration and HR). We interviewed residents from both dementia-focused and non-dementia environments — accepting that many residents in both settings lived with dementia, but that residents living in dementia-focussed environments were more likely to show behaviours that might challenge the deployment of the technology.

## Results

### Recruitment and retention

Between November 2021 and March 2022, the four selected care homes (see Table 1) ran the CONTACT programme 24/7 for 2months. Despite ending as planned, the feasibility study did not meet its pre-determined progression criteria for a full RCT.

**Fig. 2:**
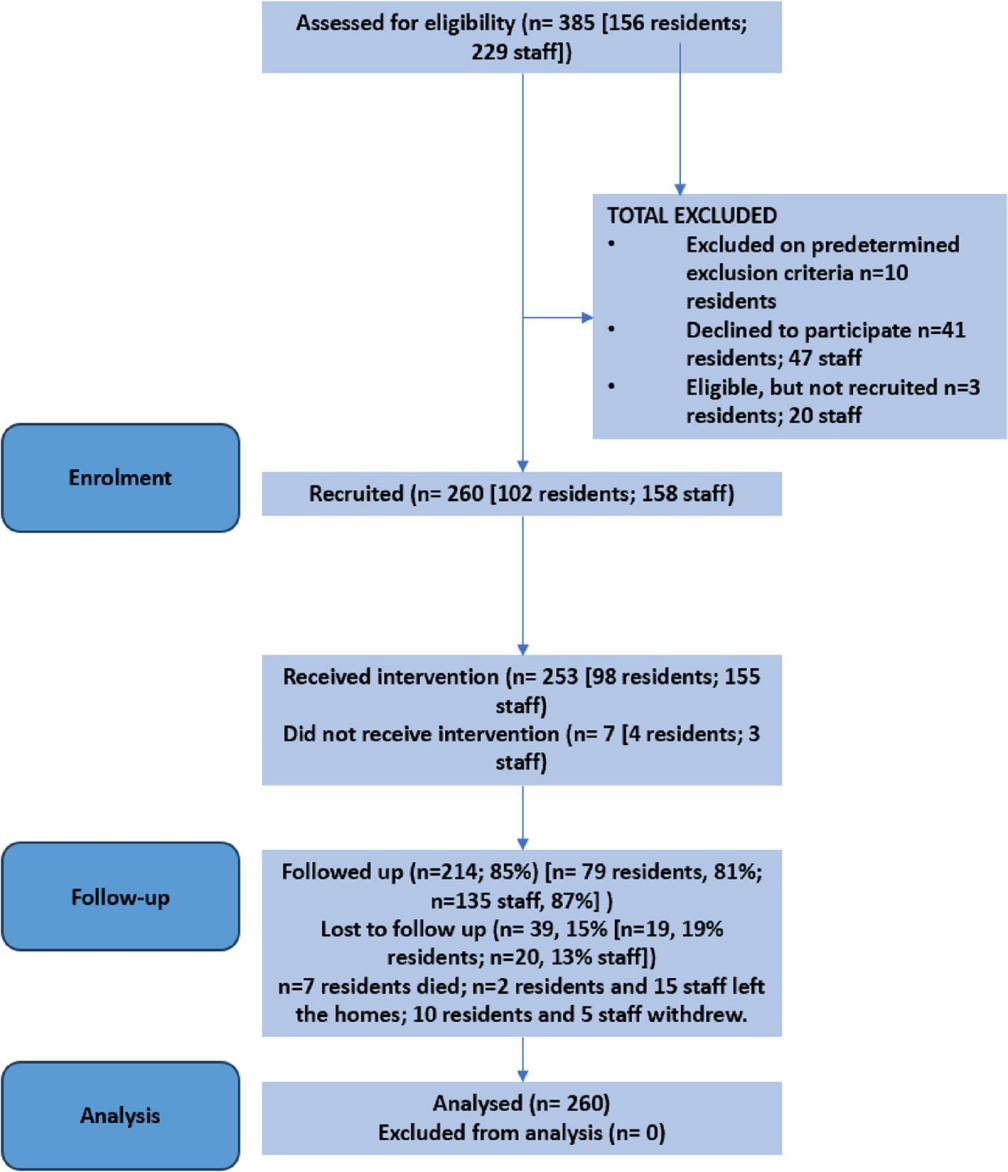
CONTACT feasibility study CONSORT diagram 28th June 2023

Of 156 screened residents (see Fig. 2), 105 consented (either personally or through a nominee) to wear a device, with 102 (97%) wearing them at the start of the 2-month intervention. Of the 225 staff deemed eligible, 82% (*n* = 178) agreed to participate, but 20 dropped out before the intervention started.

Ineligibility amongst residents was solely due to staff concerns that wearing the device could pose a risk of harm. Of the residents who declined to wear the devices, 14 did not give a reason, two were disinterested, four did not receive consent from their nominees, and two passed away before they could return their consent forms.

Of staff, 17 opted not to participate, with eight outright declining, seven not providing a reason, one objecting to wearing the device, and one simply expressing a lack of enthusiasm. Contextual factors for non-participating staff included six leaving the care home, five with imminent maternity leave, and seven categorised by managers as “rarely present” (sic.) bank staff.

The demographic profiles of the homes were female and white. Most residents had been in the homes for an extended period, and both staff and residents had been vaccinated against COVID-19. More than a third of residents lived with a dementia diagnosis (see Table 4).

**Table 4:**
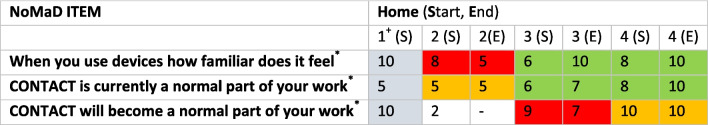
Selected adapted NoMaD scores from home managers

^*^Rated from 0 (unfamiliar) to 10 (completely familiar)

^+^No completion point data for Home 1 as home manager left before completion

Colour legend: red, less familiarity; amber, neutral; green, more familiarity

### Acceptability and feasibility of intervention delivery

#### Ease of administering devices to residents, staff, and external visitors

Getting devices to participants was moderately successful, with 70% of screened residents and 87% of staff receiving BLE wearables. But participation in CONTACT was burdensome and added to regular work. Staff highlighted screening processes, obtaining consent, and registering participants as particularly laborious. COVID-19 restrictions meant homes conducted recruitment themselves; limited digital and data infrastructure meant screening was manual and time-consuming. Larger homes bore a heavier burden; despite this, Homes 1–3 managed to complete screening on time.

Recruiting residents lacking mental capacity [31] to make decisions for themselves, and thus provide consent, meant contacting designated consultees, which further added to home workload. In some instances, the homes found the workload associated with the study outweighed the perceived benefits.

I find I have to shuffle things around to make it work. When things were heavier, I would usually finish at 5, but during the screening and consent time I had to stay late at night to contact the families. It was hard it fit it into an already hard day (Home 1, study champion).

The study’s research governance requirements contributed to CONTACT’s complexity. Every BLE wearable device’s unique number (used by the study team) needed to be cross-referenced against a “master log” in each home for the home to identify the wearer. Communications involving identifiable data were carried out via a secure file transfer system. However, university secure databases for registering participants and reporting COVID-19 cases encountered technical issues, adding further delays.

Homes 1–3 successfully dispensed devices within a month from consent and before the feasibility start date. Conversely, home four managed to issue only 66% of their BLE wearables after the study start date, with a median delay of 52.5 days (range 31, 60). Because of Home 4, the median time from consent to issuing resident devices was 33 days (range 20, 60). Several reasons were given for the 10 resident withdrawals, including residents not wanting to wear a device or feeling distressed or confused by them.

Issuing staff devices was efficient. Homes distributed them in a median of 32 days (range 12, 60). Home 4 again took longer, with a median of 35 days (range 12, 60). Reasons for staff withdrawals included no longer wanting to wear the device and finding the device irritating or inconvenient.

An original study objective was assessing the feasibility of BLE wearables for tracking visitors’ (relatives and community professionals) movements within the homes. All the homes conveyed that implementing the necessary procedures for this was not possible due to staffing constraints. Homes one and two did not have permanent reception staff, and the other homes simply judged procedures as too burdensome. Consequently, tracing visitors was dropped from study procedures.

We successfully appointed study champions in each home. Each home was informed in advance, and as part of their participation requirements, that there would be study tasks to be accommodated as work in the home. But it was clear that they struggled to absorb CONTACT-related work into non-research day to day work. Consequently, it was deprioritised by homes:

It was the time element. I don’t have an administrator or anyone else to help me with my tasks; it’s just me. CONTACT wasn’t at the top of the list by far. We said we would try our best with it, but we couldn’t (Home 3, manager and champion).

Home managers scored aspects of CONTACT familiarity, and current and future chances of “normalisation” using NoMaD (see Table 4). Managers from Homes 3 and 4 (compared to Home 2) felt more familiarity with CONTACT and that it was a more normal part of work by the end of the intervention (Home 2’s use-based familiarity diminished or stayed the same). Whilst the manager of Home 1 believed CONTACT *could* become part of normal work, they left before completing their post-implementation scoring.

Device loss and damage were noteworthy. Eleven percent of resident devices (*n* = 12) and 7% of staff devices (*n* = 7) were lost. Almost half (47%, *n* = 9) of lost or damaged devices were replaced. Fewer staff devices were lost (3%, *n* = 5) or damaged (4%, *n* = 7). Just 8% (*n* = 1) were renewed.

Fob wearables required frequent battery changes: 15% (*n* = 38) in Homes 3 and 4. These were supposed to be done by the homes, but Home 4’s delays meant a research team member undertook these over two visits. Card wearables in Homes 1 and 2 required no battery changes.

#### Acceptability and feasibility of structured CONTACT feedback

**Fig. 3:**
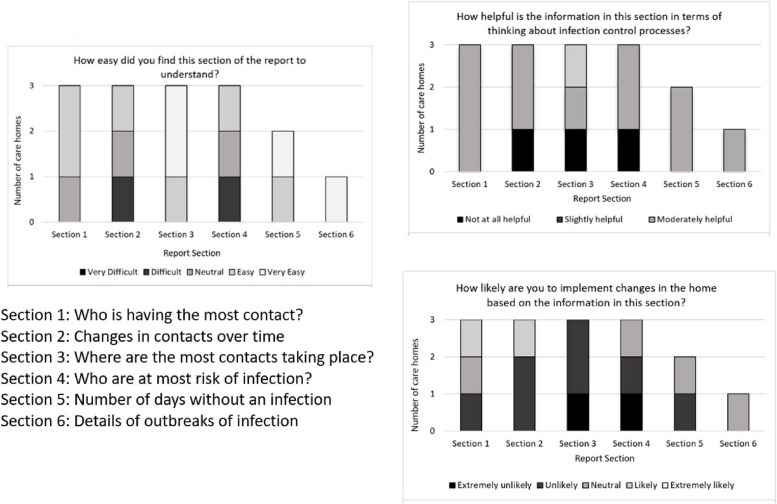
Managers’* assessed understandability, IPC influence, and change likelihood — structured reporting. *Home 3 did not provide post-scheduled report data

Home (1, 2, and 4) managers provided assessments of the (i) understandability; (ii) influence on IPC thought, and (iii) likelihood of changes based on the report (Fig. 3).

No clear patterns were evident in the assessment of report sections (see Fig. 3) by home managers. Only two homes (2 and 4) provided a judgement on structured report Sects. 5 and 6, and only Home 4 provided an assessment of report Sect. 6. For Homes 1, 2, and 4, the reports were unlikely to lead to change. Only one home (Home 1) was ambivalent (neutral) towards Sects. 1 and 2. And all 3 completing homes viewed Sect. 3 as most unlikely to induce any change.

The quantified assessment of CONTACT’s inability to induce change was evident in qualitative findings. CONTACT’s research study context, and delivery alongside competing pressures such as maintaining staffing and pre-existing infection prevention and control (IPC) requirements, diminished the perceived value of the study’s information, contributing to an overall perception that the study was of limited value:

The triggered report covered mostly what we knew already. The scheduled report identified which residents are most at risk, but what can you really do with that information? We can make people isolate but then you lose staff. The staff do a lateral flow test before work every morning, that’s the protection we already have without losing too many staff (Home 4, study champion).

…it could work, preventing us having to close because we’ve got 2 cases out of 80 for any infection. We can easily isolate pockets of people if we needed to and staff as well. So, I can see if we didn’t have the national guidelines in place, where it would give me research-based information to make risk assessment decisions…. In the guidelines, it does say that registered managers are accountable for decisions. Outside of a trial, it would have given me the confidence to say this is what the infection is doing, and we can safely isolate that and carry on doing what we are doing with the other residents, so the residents don’t suffer from lack of visitors (Home 4, manager).

A significant barrier to feasibility, reducing trust and study compliance was staff concern at “being tracked”. As a result, scheduled reports were not shared by Home 4’s management with other staff. Reports were disseminated in the other homes. The follow-up support call from researchers after each report was perceived as highly beneficial by managers and champions.

Delivering CONTACT required training for study champions and home staff. Of the 34 individuals invited to attend virtual training across 9 sessions, almost two-thirds (65%) participated (Table 5).

**Table 5:** CONTACT training session attendance

Home	Invited	Attended	Percentage
1	9	3	33%
2	4	4	100%
3	7	5	71%
4	14	10	71%
**Total**	**34**	**22**	**65%**

### Acceptability and feasibility of study design/implementation processes

Despite securing the necessary ethical and research governance approvals, we were unable to link residents in the homes to NHS (National Health Service) data. Dialogue with NHS Digital began a year before the intervention period, but linkage proved impossible in the timeframe. DSHC infection and mortality data for the homes was eventually secured, *after* the intervention period.

### Data capture

**Fig. 4:**
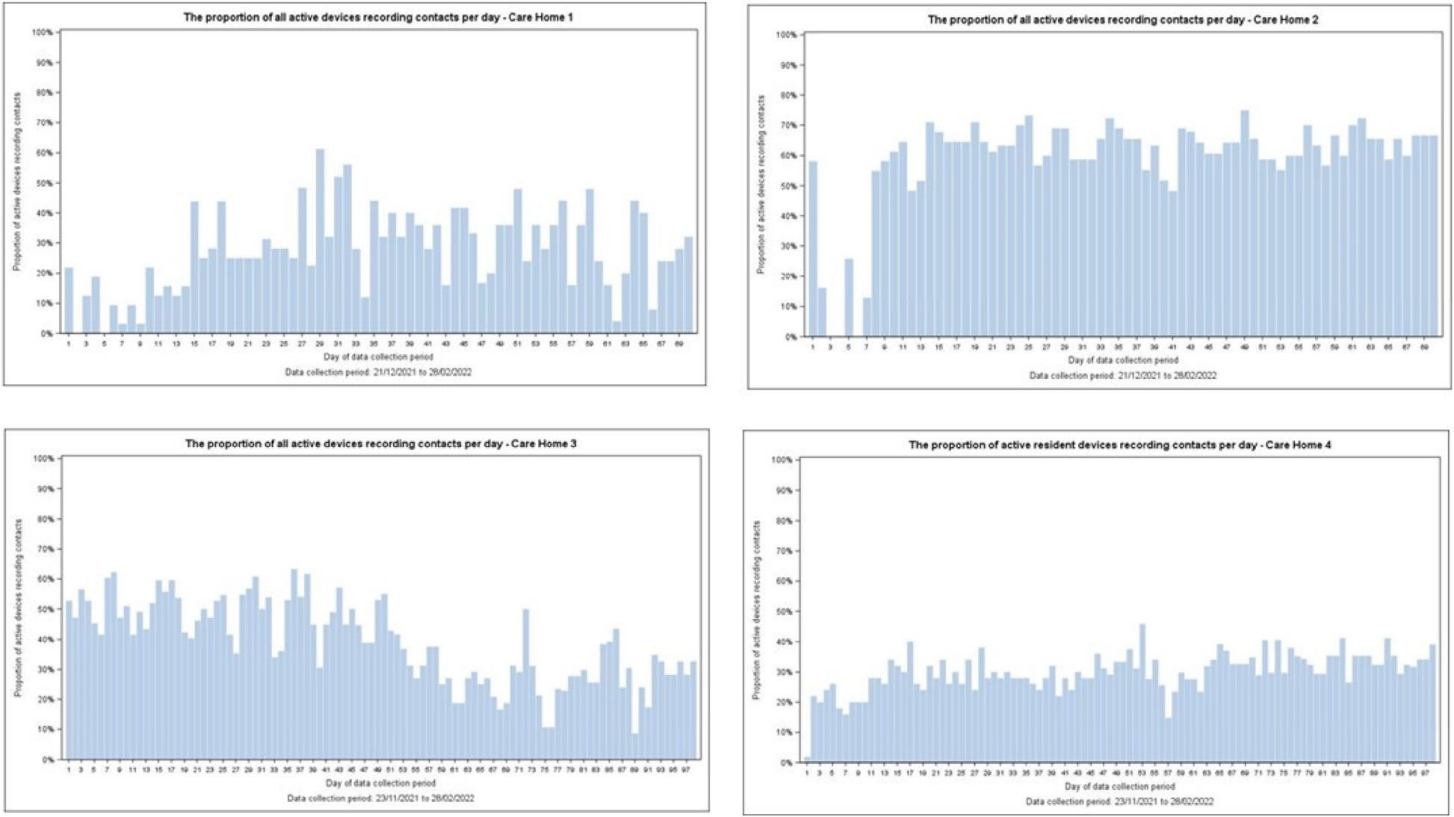
Proportion of active devices correctly recording per day by home

**Fig. 5:**
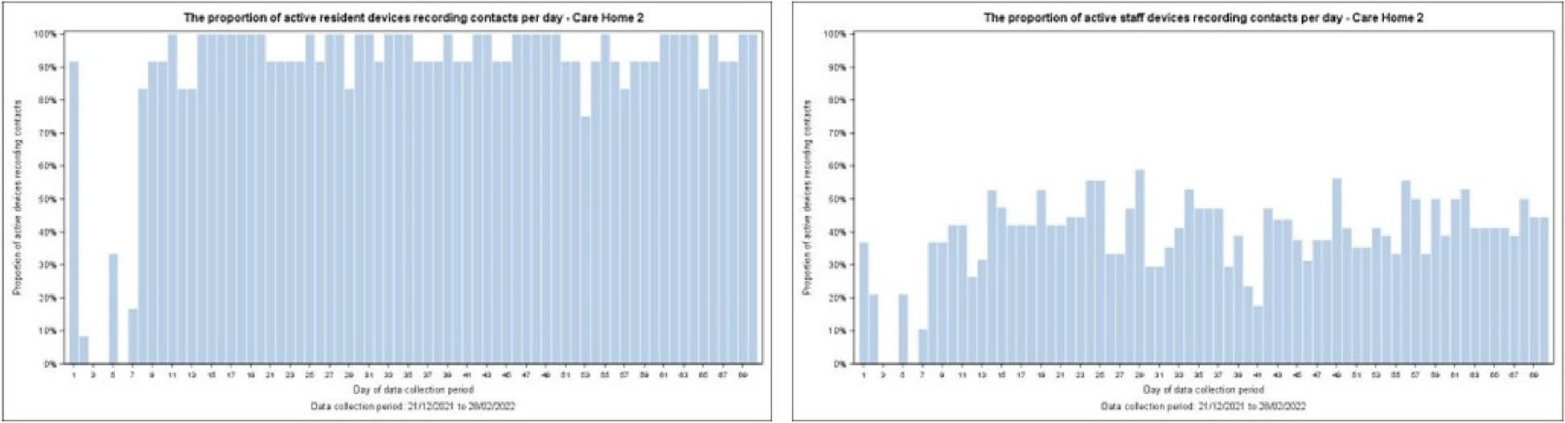
Proportion of active devices correctly recording for residents and staff — Home 2

Only around 29% (*n* = 70) of devices functioned as expected, with only minor differences between resident (29%) and staff (28%) devices. Differences between (Fig. 4) homes were evident: more day-to-day variability in Homes 1 and 3; relatively stable adoption in Homes 3 and 4; a visible dis-adoption trend in Home 3. Within Home 2 (Fig. 5), resident data was relatively complete and stable, but staff data was partial, variable, and notably absent for a short time early in the implementation period. Apparent device malfunction could be due to battery failure, inappropriate device placement, or staff not updating weekly logs for active devices — a crucial element for correctly processing data. Data transmission from our commercial partner to the university’s secure database experienced no issues.

During the feasibility period, 33 (32%) of 102 residents and 53 (34%) of 158 staff reported COVID-19 infections, suggesting self-reported COVID-19 was a feasible primary outcome. However, the single reported case of staff gastroenteritis suggests “other infections” was a less feasible outcome. Although all homes provided reported deaths (*n* = 7, 7%) during the intervention, only two homes (3 and 4) shared data regarding whether the deaths were COVID-19 related and the months from registration or device issue to death. Despite 86 infection notifications, only 52 (60%) contact reports were requested by the homes.

### Progress against predefined criteria

The study did not meet any of our quantitative criteria for progression to a definitive RCT. Additionally, qualitative data from the homes indicated study demands were too burdensome and excessive. Projected compliance and participation rates were too low to justify a definitive trial (Table 6).

**Table 6:**
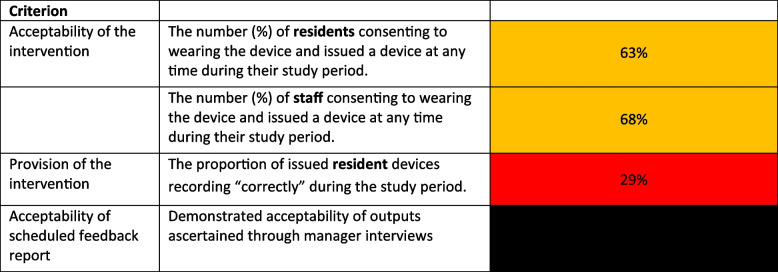
Progression criteria achievement

## Discussion

A definitive trial of the CONTACT intervention using BLE wearables and feedback to homes for improved IPC decisions, at least in a pandemic context, was unfeasible. The intervention’s development, implementation, and evaluation were executed during the COVID-19 pandemic, a contextual factor that significantly reduced the feasibility of the intervention.

The planning and development process was hastily executed, leading to a lack of proper adaptation for a care home context. For instance, BLE fob devices required cleaning when exposed to human waste or food. More and longer co-produced planning could have promoted design adjustments [32]. Peryer and colleagues’ synthesis of studies of contextual factors influencing complex intervention research makes some key recommendations to enhance the chances of successful intervention delivery and reduce “procedural drift” [33]. Our use of NPT to frame intervention development (and understand the processes of CONTACT implementation) [16] meant our protocol addressed most of the recommendations. For example, we discussed integration into existing work, adapted and simplified data collection, etc. But still CONTACT — in blunt terms — was not feasible. The key was in the need for “authentic” (sic.) [33] co-production. The pre-vaccine availability context of intervention development meant that haste and speed were important and pragmatic choices dominated. The post vaccine context of in situ implementation perhaps meant that we could have spent more time on co-production of key aspects such as dashboard presentation and increasing managerial “pull” for CONTACT’s “pushed” information. The remote nature of implementation hindered research team, “supply side”, control, and adaptation (to changing context) speed. Implementing CONTACT and study procedures was primarily carried out by the care homes, with minimal in-person support from the research team due to pandemic-related restrictions. They did not have the capacity for this implementation work. Most specifically, the study champion role required dedicated time for successful enactment of wearable-related work in a rigorous research study and implementation context.

We used Normalisation Process Theory (NPT) at planning and implementation stages to mitigate some of these effects, but its utility was limited in the pressing circumstances of the pandemic [34]. The intervention demanded additional work from care homes already struggling with everyday care. CONTACT’s perceived benefits did not sufficiently outweigh pre-existing methods of IPC, limiting its appeal [35]. The idea of rectifying an information deficit through BLE wearable data and analysis only has merit if information does not come with too high a cost [36]. Like other aspects of health and social care, high-quality tailored information does not always lead to informed choices [37]. The “pull” for the information we were “pushing” [38] was further diminished by the — albeit welcome — development of a successful vaccination programme for COVID-19.

Technical issues were also a barrier. BLE wearables rely on RSSI signal strength to determine proximity and potential exposure. RSSI can be distorted by physical barriers or other device interference, reducing accuracy [12, 39, 40]. Further, real-world implementation issues led to suboptimal procedure compliance and low population coverage.

As with others’ experiences of tech-enabled contact tracing, privacy was a significant hurdle to implementation [41]. The tracking ability of the technology was seen as intrusive, undermining trust in the technology and IPC amongst staff. CONTACT was designed to offer insight into staff interaction times and movements. This ability to make staff “visible” deterred adoption. Australian care home research suggest limited interactions may make invisibility more desirable than is sometimes assumed [42, 43]. Until such privacy concerns can be adequately addressed, the widespread use of wearable technology with tracking and tracing capabilities in care homes remains unlikely.

The success of BLE wearables for contact tracing hinges on consistent use and device maintenance by individuals. In care homes, where many residents have cognitive and physical limitations, staff support is crucial. However, staff found the devices intrusive and burdensome. This crucial [35] lack of added value or perceived advantage reduced adoption: unwillingness to encourage residents to participate in the CONTACT study and wear the devices.

CONTACT faced a 12-month delay waiting for the permissions from the UK’s Social Care Research Ethics Committee to deliver CONTACT as part of “care as usual” — given the pandemic context. Despite gaining the required permissions, care homes insisted on individual consent procedures, citing fears of punitive action from the Care Quality Commission or litigation risks. These concerns, though unfounded, are indicative of a broader tendency to utilise administrative procedures to mitigate perceived risks — even if such actions might inadvertently compromise care quality [44]. They also reflect a wider failure to support care homes’ research readiness, despite rhetoric from national research funders to the contrary [45].

The movement of people into and between care homes was a significant factor in the spread of COVID-19 [46, 47]. The burden associated with the CONTACT study, staff restrictions, and infrastructural deficiencies made it impossible to extend the technology to visitors, thereby missing a key source of potential infection tracing.

Although we provided CONTACT’s technology to homes free of charge, there were associated costs such as data management, analysis, and technical support for system installation, battery changes, and replacement devices. Given the perceived lack of value, it seems unlikely that care homes would be willing to absorb these costs or pass them onto the purchasers of care.

To effectively utilise the information generated by BLE wearables, staff need a degree of information literacy to understand concepts like individualised risk and infection trends. Limited numeracy and information skills can be a barrier to innovation in care homes [48]. Managers suggested CONTACT’s structured reporting used in CONTACT was difficult to comprehend, contributing to the perception that they were unlikely to use the information as a basis for change. This was compounded by a lack of trust in the results amongst some staff.

### Implications for future research

CONTACT was unfeasible in a pandemic context. Nonetheless, digital contact tracing systems still have promise, albeit based on low-quality evidence from modelling and simulation studies [12, 49]. The implication is that effective implementation is a key determinant of successful contact tracing and improved infection prevention and control (IPC), not the technical efficacy of BLE wearables [40].

Future research involving BLE wearable systems should concentrate on applying known strategies for successful research with care homes [32] and dedicating time to co-produce BLE wearable systems that minimise the burden for participating homes. Facilitators such as privacy, trust, and the utilisation of valuable data from such systems should be a focus of planning and implementation. Pragmatic choices will always be inevitable in future epidemic contexts, but in the same way that theory (NPT in our case) helps understand *why* an intervention may not be feasible, existing synthesis of empirical studies of responses to contextual factors could provide a practical start point for informed, but rapid, intervention development and implementation planning with care homes [33].

As with any new intervention, learning and refinement through evaluation — even of failure — are key. To maximise this learning, the use of appropriate theories of implementation, innovation adoption, and decision-making can ensure that failures contribute to broader literature and efficient intervention development. In this context, hybrid studies that combine an implementation focus with measuring effectiveness could yield the most valuable insights [50].

### Limitations

CONTACT had several limitations. Firstly, not all staff and residents who wore the technology took part in the feasibility assessment. Positive views of the intervention may have been missed. Additionally, key staff members involved in the study, notably the manager in Home 1, left during the feasibility assessment, destabilising the home and impacting study implementation.

Another constraint was the limitation on the research team’s presence in the care homes due to COVID-19 restrictions. Our development, implementation, and evaluation processes were largely conducted remotely and virtually, negatively impacting on these critical study aspects.

With the easing of restrictions and more time to focus on building relationships during the development, delivery, and evaluation of an intervention, it is conceivable that a CONTACT-style intervention may prove more feasible in the future (Table 7).

**Table 7:** CONTACT feasibility study objectives and outcomes

Study objective	Data collection method/outcomes
**Assess the acceptability and feasibility of intervention delivery processes**	
Contact tracing devices	
Evaluate ease of device administration	5-point Likert scale question(s) measuring ease of use/administration of devices at end of study period
Evaluate feasibility of data collection linking devices with individual identities for residents, staff, and visitors	completion levels of resident, staff, and visitor wear logs detailing device ID, weekly
Explore acceptability of wearing devices and reasons for non-wear	Percentage of participants wearing the device (for the duration of the study) and reasons for non-wear
Explore loss/breakage/replacement requirements in a 1-month period	Number (percentage) of active devices lost/broken/replaced reported in device wear log
***Tailored feedback***	
Explore feasibility of proposed methods of CONTACT tracing feedback (format, content, frequency)	Interviews aimed at understanding and usability of feedback, alongside expressed preferences for content, frequency, and format of the feedback
Evaluate research team processes and capacity for handling queries/problems from homes relating to intervention delivery	Logs detailing the number and nature of queries from each site and the time taken to resolve queries
***Site engagement — intervention delivery***	
Explore barriers to study champion role in the homes	Interviews focused on study role, potential barriers, and levers
Attendance and engagement with face-to-face training for the champion and CONTACT device use understanding	Personnel attending vs expected. CRF (Case Report Forms) checklist for the delivery of training elements, details of any changes to training and reasons why, and understanding of key learning objectives
Evaluate feasibility of support phone calls to (intervention) homes	Researcher-completed call logs detailing frequency and number (percentage) of successful phone calls completed for each site, call length and reasons for calls not taking place
Attendance and engagement with training webinars	Webinar logs completed by training provider, collecting the number of attendees at each webinar
**Assess the acceptability and feasibility of study design/implementation processes**	
*** Device software***	
Evaluate success/failure in data capture, transmission, and analysis as well as rates of contacts and reasons behind the data-driven picture	Completion of resident, staff, and visitors’ logs cross-checked with flagged data from a random sample (resident/staff) of contact tracing reports to ensure appropriate data capture with documented reasons for missing data (i.e. resident bed-bound/staff leave)
Ensure data transmission software works (reading of transferred data at trials unit; storage; analysis)	Verification of data retrieved from MicroShare against list of devices known to be sent to home. For each device to be recording data “correctly” it needed to be issued, not showing a continuous contact of > 6 h and to have at least one additional contact in a day. Thus, for each device, we can compare observed (data) vs. expected (data)
Investigate non-compliance/site adaptations of technology or study processes	Reports are generated to identify devices that appear inactive which can be used as an indicator of staff non-compliance at site
***Site engagement — study delivery***	
Evaluate site willingness and capacity for definitive main trial; degree of commitment to the study?	Interviews to gain feedback on participation and any potential barriers
Site issues managing the study?	Logs detailing the nature of queries will be recorded. Additional Feedback from interviews with manager/gatekeeper
Any issues from study team in delivery in the real world?	Interviews with key staff on study procedures
Feasibility of collecting (planned definitive study) primary outcome data (COVID-19 test results)	Ease of extracting data from care home records; overall number and percentage of residents we know had a COVID-19 test (minimum monthly). The number of positive COVID-19 tests out of those that had a test

## Conclusion

The CONTACT intervention of BLE wearables for contact tracing and feedback was unfeasible and unacceptable to care homes. Intervention planning, execution, and evaluation took place during the COVID-19 pandemic and coincided with the discovery of a successful vaccine against the disease. These factors influenced the research team’s methodology and the care homes’ willingness and ability to implement the intervention.

Despite these setbacks, the technology underpinning CONTACT shows promise. Consequently, future research is recommended, but with an important shift in focus: researchers should aim to co-design studies with care homes and place equal, if not greater, emphasis on the successful implementation of the intervention, rather than the technical effectiveness of the wearable devices.

## Supplementary information

Supplementary Material 1.  Additional file 1: Appendix A. Scheduled report feedback. Appendix B. Reactive “triggered” report example

Supplementary Material 2.

Supplementary Material 3.

## Data Availability

The datasets generated and/or analysed during the current study are not publicly available as data contains sensitive information for residents and families of the homes, and we cannot rule out the possibility that those close to the homes may think they recognise certain aspects of context. Anonymised Social Network data on contact patterns for four homes are available from the corresponding author on reasonable request.

## Abbreviations

BLE: Bluetooth enabled
RCT: Randomised controlled trial
RSSI: Received signal strength indicator
NPT: Normalisation Process Theory
NoMaD: Normalisation Measure Development questionnaire
NHS: National Health Services
NIHR: National Institute for Health and Social Care Research
CONTACT: CONtact TrAcing in Care homes using digital Technology
IPC: Infection prevention and control
SARS-CoV-2: Severe acute respiratory syndrome coronavirus 2
COVID-19: Coronavirus disease
IoT: Internet of Things
GPS: Global positioning system
LoRaWAN: Long-Range Wide-Area Network
ENRICH: Enabling Research in Care Homes
NICHE-Leeds: Nurturing Innovation in Care Home Excellence in Leeds
CQC: Care Quality Commission
CRF: Case report form(s)
