# Supplementary material for: CONTACT: a non-randomised feasibility study of bluetooth-enabled wearables for contact tracing in UK care homes during the COVID-19 pandemic
Source: Pilot Feasibility Stud. 2024 Oct 2;10:125. doi: 10.1186/s40814-024-01549-6 (PMC11445870; doi:10.1186/s40814-024-01549-6)
Supplement: Supplementary file 1 — Supplementary Material 1. Additional file 1: Appendix A. Scheduled report feedback. Appendix B. Reactive “triggered” report example [file 40814_2024_1549_MOESM1_ESM.docx]

**Supplementary Table 1. Consolidated criteria for reporting qualitative studies (COREQ): 32-item checklist.**

| **Topic and Item No.** | **Guide Questions/Description** | **Comment** |
| --- | --- | --- |
| **Domain 1: Research team and reflexivity** | | |
| Personal Characteristics | | |
| 1. Interviewer/facilitator | Which author/s conducted the interview or focus group? | ADR, AS |
| 2. Credentials | What were the researcher’s credentials? | BSc, PhD; BSc MSc |
| 3. Occupation | What was their occupation at the time of the study? | ADR: Senior Research Fellow  AS: Research Fellow |
| 4. Gender | Was the researcher male or female? | Female. |
| 5. Experience and training | What experience or training did the researcher have? | The researchers had extensive experience in face-to-face interviews and focus groups. They had also supervised qualitative research. |
| Relationship with participants | | |
| 6. Relationship established | Was a relationship established prior to study commencement? | ADR was involved in the recruiting of the care homes and initial discussions with the management of the participating homes. |
| 7. Participant knowledge of the interviewer | What did the participants know about the researcher? | Participants knew about the researchers‘ affiliation. |
| 8. Interviewer characteristics | What characteristics were reported about the interviewer/facilitator? | None. |
| **Domain 2: Study design** | | |
| Theoretical framework | | |
| 9. Methodological orientation and Theory | What methodological orientation was stated to underpin the study? | Normalisation Process Theory [NPT] and interpretive evaluation. |
| Participant selection | | |
| 10. Sampling | How were participants selected? | Pragmatically (homes); purposively by role (staff); pragmatically (residents) |
| 11. Method of approach | How were participants approached? | face-to-face and facilitated by home staff (residents). |
| 12. Sample size | How many participants were in the study? | 38 interviews with 33 participants. |
| 13. Non-participation | How many people refused to participate or dropped out? Reasons? | N/A. |
| Setting | | |
| 14. Setting of data collection | Where was the data collected? | Workplace and virtually via Teams/Zoom. |
| 15. Presence of non-participants | Was anyone else present besides the participants and researchers? | No. |
| 16. Description of sample | What are the important characteristics of the sample? | Four care homes (30-102 residents), all “for-profit” and rated “good” by CQC . Residents: mean age 86.1 years (SD 8.5); 73% female; 100% white; median 99 weeks in the home (range 2-590); 37% with a diagnosis of dementia; 20% tested positive previously for COVID-19.  Staff: mean age 42.1 years (SD 14.7); 87% female; ethnicity n/a; 26% previously COVID-19 +; employed 123 median weeks (range 0 – 1302 weeks); 90% permanent staff; 64% care or nursing staff; less than 1% worked in more than one home.  Both: 99% vaccination rate. |
| Data collection | | |
| 17. Interview guide | Were questions, prompts, guides provided by the authors? Was it pilot tested? | The overall interview topics were reported. The interview guide was pilot tested in a non-participating nursing home. |
| 18. Repeat interviews | Were repeat interviews carried out? If yes, how many? | Yes, five (3 manager/champions and 2 champions) start and end of intervention. |
| 19. Audio/visual recording | Did the research use audio or visual recording to collect the data? | Data were audio recorded using a digital recorder or captured as part of virtual platform. |
| 20. Field notes | Were field notes made during and/or after the interview or focus group? | yes. |
| 21. Duration | What was the duration of the interviews or focus group? | Telephone interviews: 25 minutes on average |
| 22. Data saturation | Was data saturation discussed? | Data saturation was discussed but the nature of the care home context and time frames associated with data collection and the intervention and non-grounded theory approach adopted meant it was not appropriate in this study. |
| 23. Transcripts returned | Were transcripts returned to participants for comment and/or correction? | No. |
| **Domain 3: Analysis and findings** | | |
| Data analysis | | |
| 24. Number of data coders | How many data coders coded the data? | 2 (ADO, AS) + 1 overview and check (CT). |
| 25. Description of the coding tree | Did authors provide a description of the coding tree? | The coding tree start point were the four core constructs of NPT that were developed abductively. |
| 26. Derivation of themes | Were themes identified in advance or derived from the data? | On the basis of the abductive coding stemming from NPT, themes were interpretatively derived from the data. |
| 27. Software | What software, if applicable, was used to manage the data? | MS Excel . |
| 28. Participant checking | Did participants provide feedback on the findings? | No. |
| Reporting | | |
| 29. Quotations presented | Were participant quotations presented to illustrate the themes / findings? Was each quotation identified? | Participant quotations were presented. Each quotation was identified by referring to the care home and interviewee role in the home. |
| 30. Data and findings consistent | Was there consistency between the data presented and the findings? | Yes. |
| 31. Clarity of major themes | Were major themes clearly presented in the findings? | Yes. |
| 32. Clarity of minor themes | Is there a description of diverse cases or discussion of minor themes? | Yes. |
