# Supplementary material for: CONTACT: a non-randomised feasibility study of bluetooth-enabled wearables for contact tracing in UK care homes during the COVID-19 pandemic
Source: Pilot Feasibility Stud. 2024 Oct 2;10:125. doi: 10.1186/s40814-024-01549-6 (PMC11445870; doi:10.1186/s40814-024-01549-6)
Supplement: Supplementary file 2 — Supplementary Material 2. [file 40814_2024_1549_MOESM2_ESM.pdf]

What does your CONTACT data show?

REPORT 3 - 18/02/2022

Here are contacts* between residents, staff and visitors in your home since the previous report. This report highlights who, where and when contacts are happening and how contacts are changing over time. It also provides information on any infections occurring in your home.

CONTACT team researchers will call you in a few days to talk through the information. They can help you understand what, if any, action you might like to consider.

**CONTACT is two people within 2 metres for 15 minutes or more*

Residents with the most contacts

15 (83%) of residents had contact with **another resident**

17 (94%) of residents had contact with **a staff member**

32 (82%) of staff had a contact with **a staff member**

Device ID	Resident	Staff	Visitor
AC233FA8BD68	18	26	0
AC233FA8BD23	15	22	0
AC233FA8BD1A	10	26	0
AC233FA8BD2B	13	22	0
AC233FA8BD0C	12	20	0
AC233FA8BCFB	14	16	0
AC233FA8BD17	10	20	0
AC233FA8BCFA	8	18	0
AC233FA8BD1C	9	14	0
AC233FA8BD1F	12	10	0

Most contacts — Least contacts

Changes in contacts over time

The bars and lines on these graphs show different information. The bars show the total number of unique device pairings each week. Example: Device A records contact with Device B three times, Device A records contact with Device C five times, Device B records contact with Device D six times - the unique number of pairings is three.

The dots/lines in the graph show the average number of contacts that each device has had in one week. Example: like before, Device A records contact with Device B three times, Device A records contact with Device C five times, Device B records contact with Device D six times. The average is calculated by dividing the total number of contacts each device has had - 28 - by the number of devices - 4. So, the average number of contacts is 7

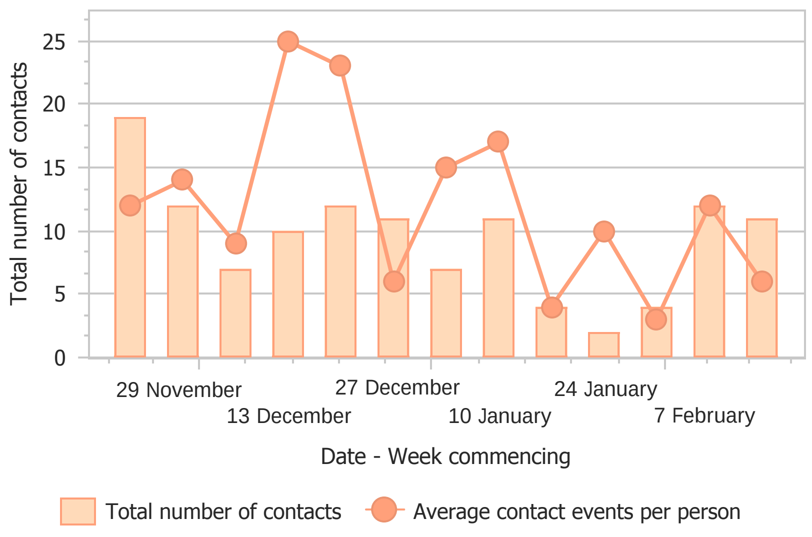

Resident - Resident

Key Message

The total number of pairs of resident-resident contacts each week has remained relatively stable with a dip towards the end of January.

The average number of contacts per resident has fluctuated over time showing no particular pattern.

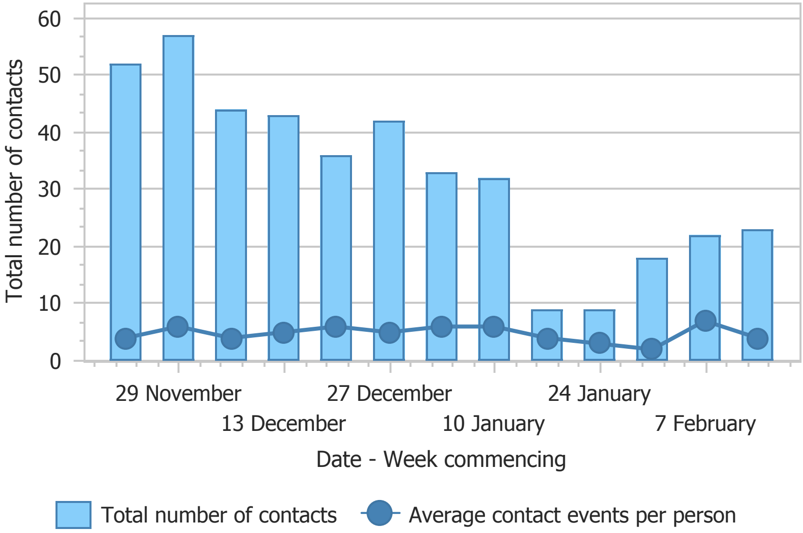

Resident - Staff

Key Message

The total number of pairs of resident-staff having contact each week showed a steady decline until end January when it started to increase again.

The average number of times residents have had contacts with staff has however remained relatively stable.

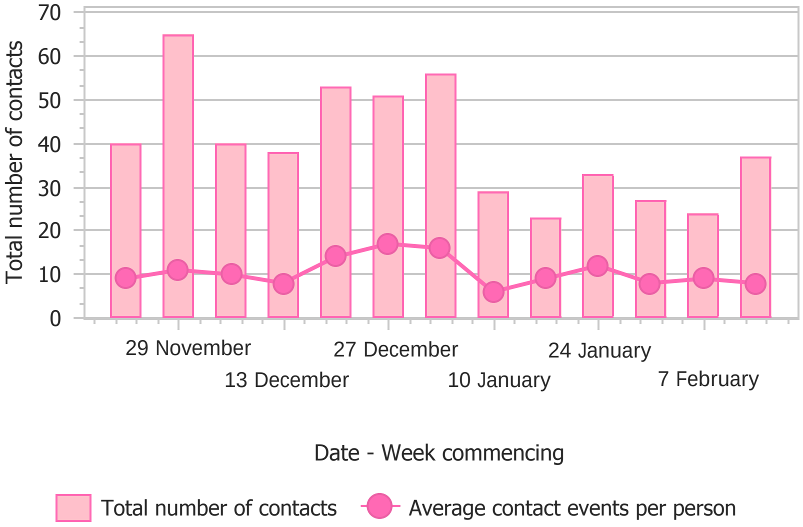

Staff - Staff

Key Message

The total number of pairs of staff-staff having contact with each other has remained relatively stable since the beginning of January. The average number of times in a week that staff are having contact with each other has also remained reatively stable at around 10 contacts each per week.

Where are the most contacts taking place?

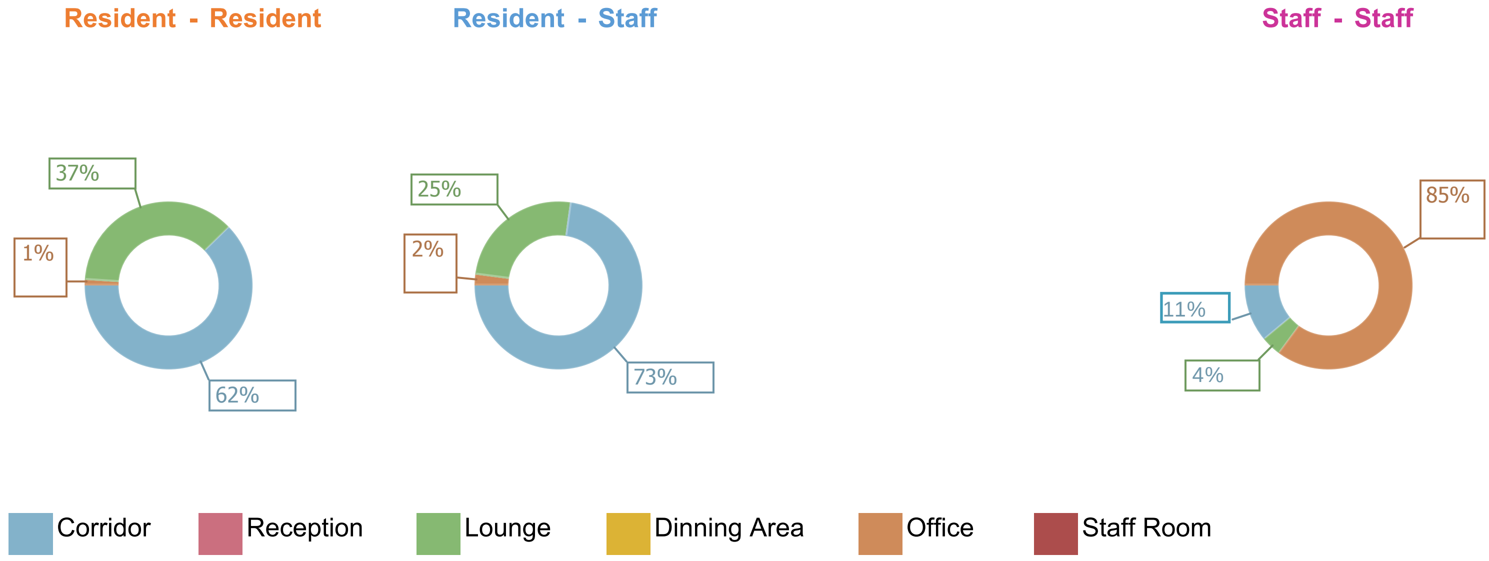

Who are at most risk of infection?

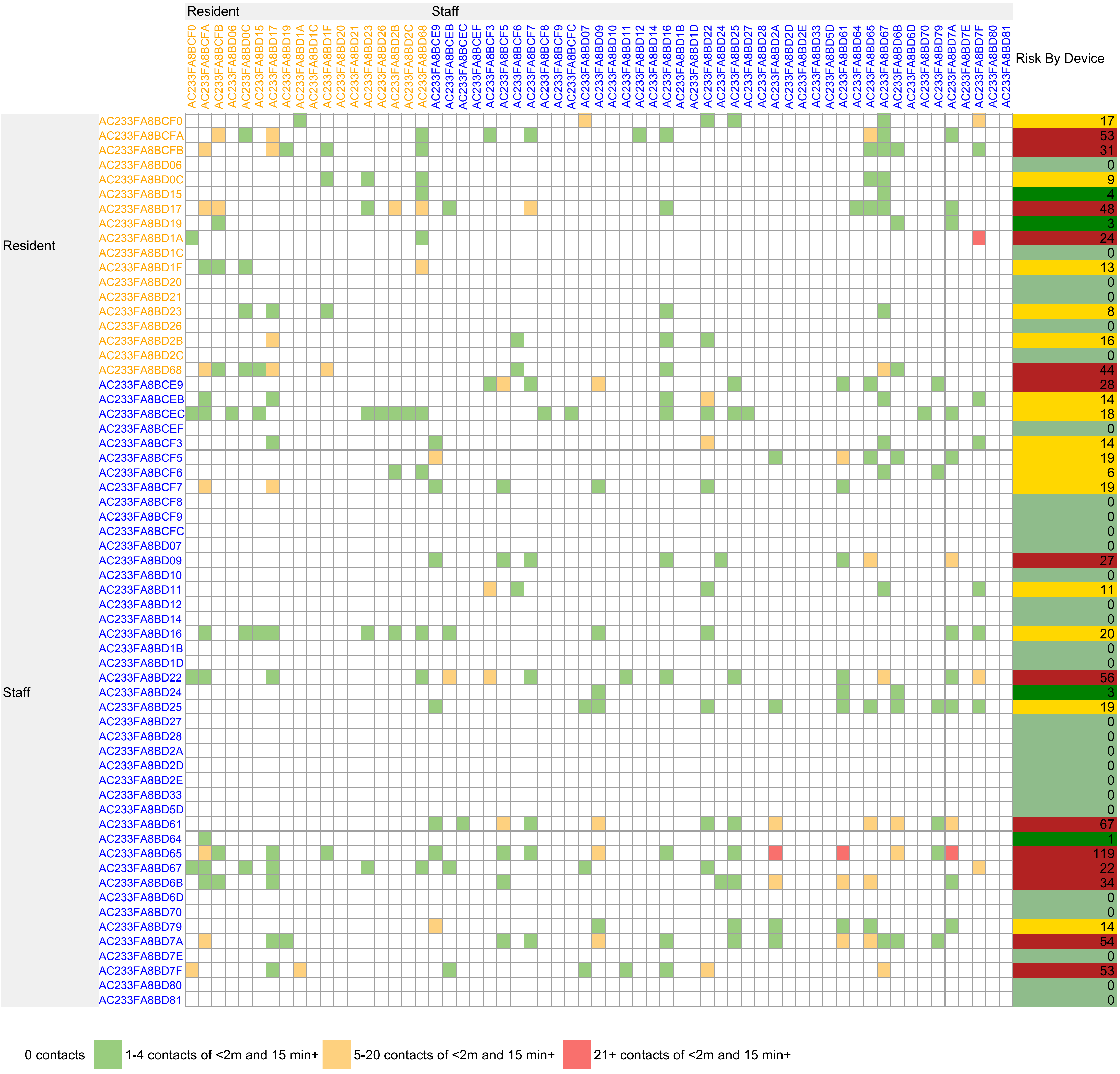

Group	Device	Risk By Device
Resident	AC233FA8BCF0	17
Resident	AC233FA8BCFA	53
Resident	AC233FA8BCFB	31
Resident	AC233FA8BD06	0
Resident	AC233FA8BD0C	9
Resident	AC233FA8BD15	4
Resident	AC233FA8BD17	48
Resident	AC233FA8BD19	3
Resident	AC233FA8BD1A	24
Resident	AC233FA8BD1C	0
Resident	AC233FA8BD1F	13
Resident	AC233FA8BD20	0
Resident	AC233FA8BD21	0
Resident	AC233FA8BD23	8
Resident	AC233FA8BD26	0
Resident	AC233FA8BD2B	16
Resident	AC233FA8BD2C	0
Resident	AC233FA8BD68	44
Staff	AC233FA8BCE9	28
Staff	AC233FA8BCEB	14
Staff	AC233FA8BCEC	18
Staff	AC233FA8BCEF	0
Staff	AC233FA8BCF3	14
Staff	AC233FA8BCF5	19
Staff	AC233FA8BCF6	6
Staff	AC233FA8BCF7	19
Staff	AC233FA8BCF8	0
Staff	AC233FA8BCF9	0
Staff	AC233FA8BCFC	0
Staff	AC233FA8BD07	0
Staff	AC233FA8BD09	27
Staff	AC233FA8BD10	0
Staff	AC233FA8BD11	11
Staff	AC233FA8BD12	0
Staff	AC233FA8BD14	0
Staff	AC233FA8BD16	20
Staff	AC233FA8BD1B	0
Staff	AC233FA8BD1D	0
Staff	AC233FA8BD22	56
Staff	AC233FA8BD24	3
Staff	AC233FA8BD25	19
Staff	AC233FA8BD27	0
Staff	AC233FA8BD28	0
Staff	AC233FA8BD2A	0
Staff	AC233FA8BD2D	0
Staff	AC233FA8BD2E	0
Staff	AC233FA8BD33	0
Staff	AC233FA8BD5D	0
Staff	AC233FA8BD61	67
Staff	AC233FA8BD64	1
Staff	AC233FA8BD65	119
Staff	AC233FA8BD67	22
Staff	AC233FA8BD6B	34
Staff	AC233FA8BD6D	0
Staff	AC233FA8BD70	0
Staff	AC233FA8BD79	14
Staff	AC233FA8BD7A	54
Staff	AC233FA8BD7E	0
Staff	AC233FA8BD7F	53
Staff	AC233FA8BD80	0
Staff	AC233FA8BD81	0

Number of days without an infection

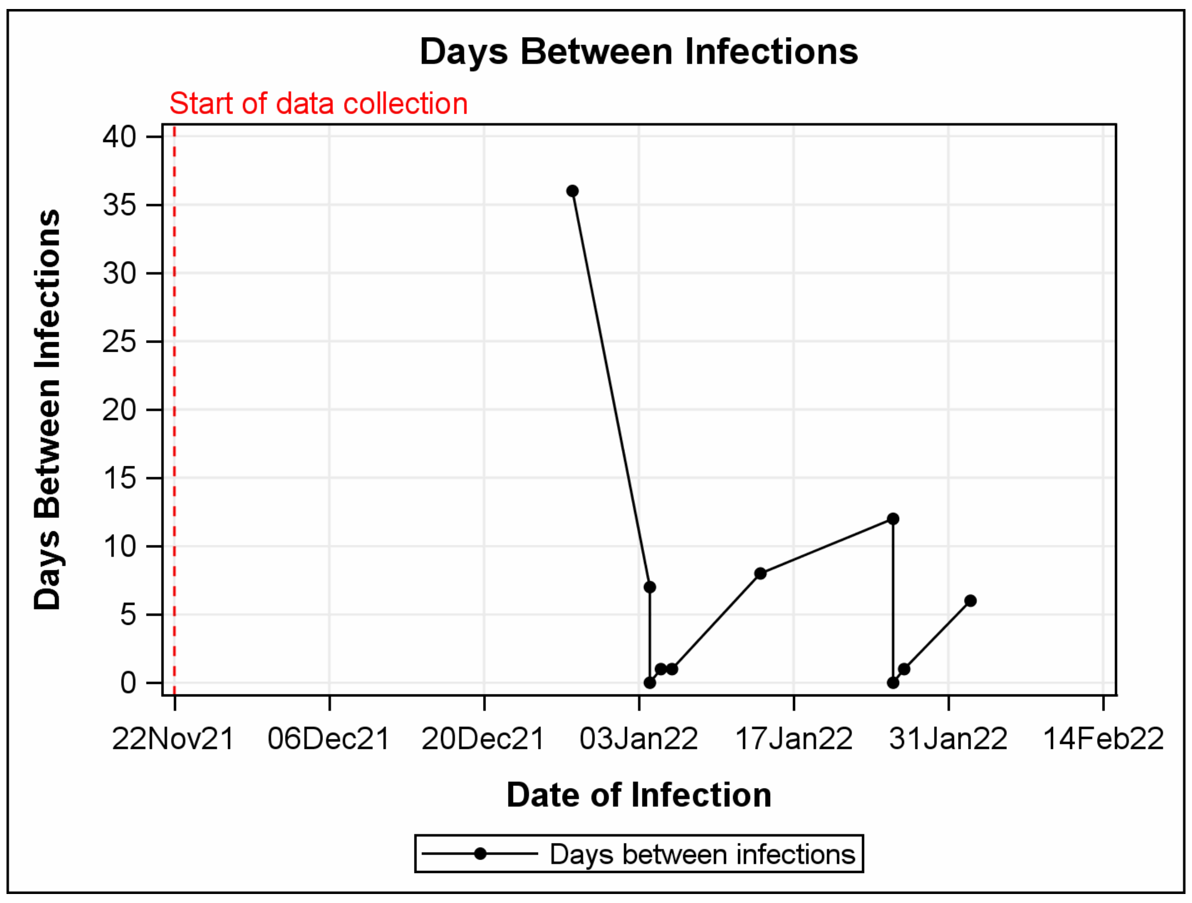

Key message

Since the start of data collection you had over 35 days before your first reported infection. However, it has been less than 14 days between subsequent infections. The goal is to increase the number of days without an infection.
