# Supplementary material for: CONTACT: a non-randomised feasibility study of bluetooth-enabled wearables for contact tracing in UK care homes during the COVID-19 pandemic
Source: Pilot Feasibility Stud. 2024 Oct 2;10:125. doi: 10.1186/s40814-024-01549-6 (PMC11445870; doi:10.1186/s40814-024-01549-6)
Supplement: Supplementary file 3 — Supplementary Material 3. [file 40814_2024_1549_MOESM3_ESM.pdf]

CONTACT

Contact tracing in care homes using digital technology

University of Leeds

Triggered Report

For the period 01/02/2022 – 03/02/2022

The CONTACT tracing technology currently in your care home transmits data relating to the number of contacts made in the home – a contact is when 2 or more devices come into contact with each other for 15 minutes or more at a distance of 2 metres or less.

This report provides you with a breakdown of those contacts.

The first page/s provide you with a general 'at a glance' quick summary of the headlines. It tells you who has come into contact with who, how many times and where. This type of information can help you to identify 'hot' areas where a lot of people might be coming into close contact with each other. The device IDs are already matched up to residents, staff and visitors. You can use your Master Device Log to find out more specific information about which device belongs to which person.

The 'detailed report' provides a more detailed breakdown of the 'at a glance' summary. This tells you about who came into contact with who, how long the contact was, what time the contact started, when it ended, and where the contact took place.

A quick reminder on how to interpret the report:

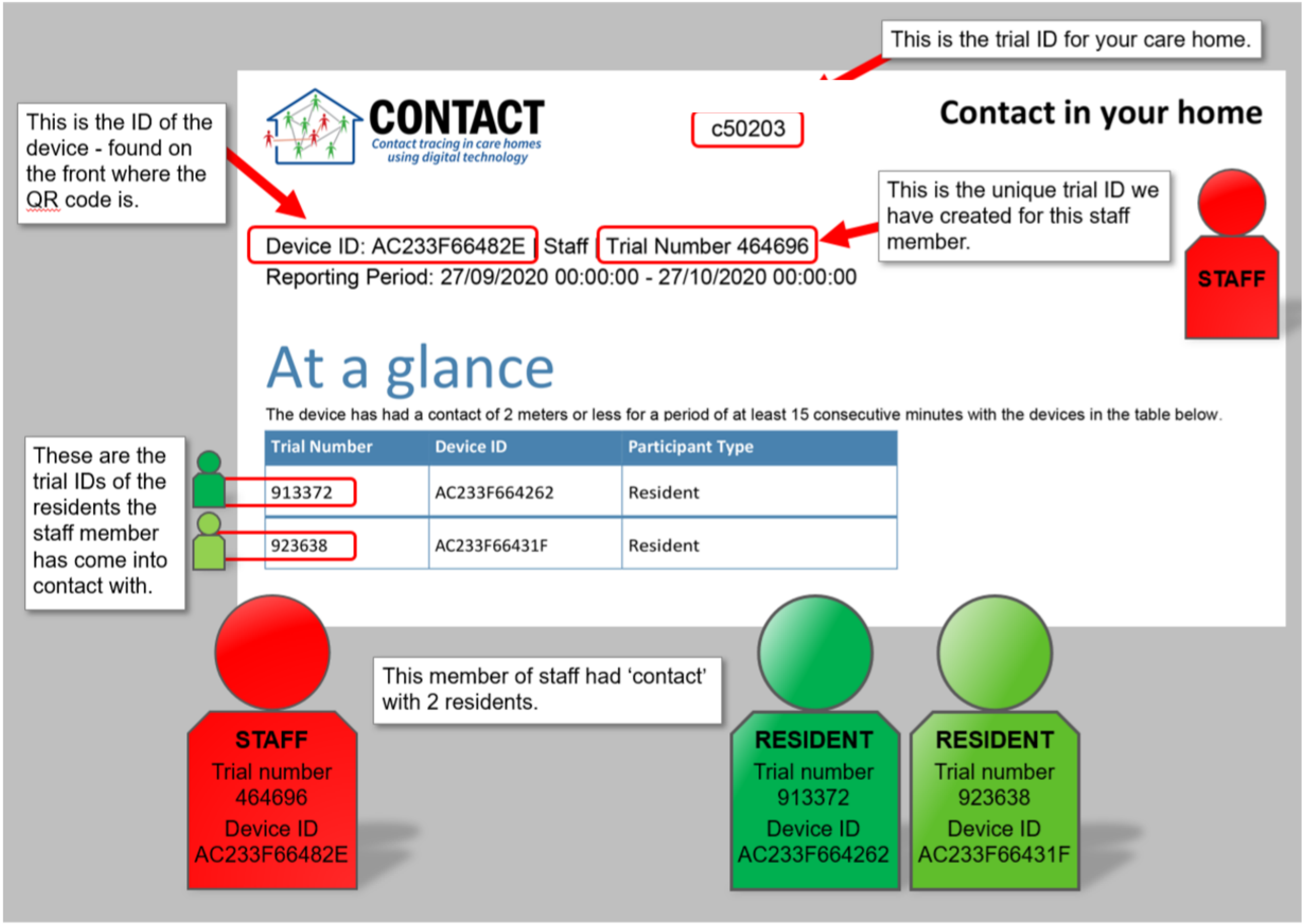
This is the trial ID for your care home.
This is the ID of the device - found on the front where the QR code is.
CONTACT
Contact tracing in care homes using digital technology
c50203
Contact in your home
This is the unique trial ID we have created for this staff member.
STAFF
Device ID: AC233F66482E Staff Trial Number 464696
Reporting Period: 27/09/2020 00:00:00 - 27/10/2020 00:00:00
At a glance
The device has had a contact of 2 meters or less for a period of at least 15 consecutive minutes with the devices in the table below.
Trial Number
Device ID
Participant Type
913372
AC233F664262
Resident
923638
AC233F66431F
Resident
These are the trial IDs of the residents the staff member has come into contact with.
This member of staff had 'contact' with 2 residents.
STAFF
Trial number 464696
Device ID AC233F66482E
RESIDENT
Trial number 913372
Device ID AC233F664262
RESIDENT
Trial number 923638
Device ID AC233F66431F

This view has the contacts that were summarised before, now separated out into each individual contact.

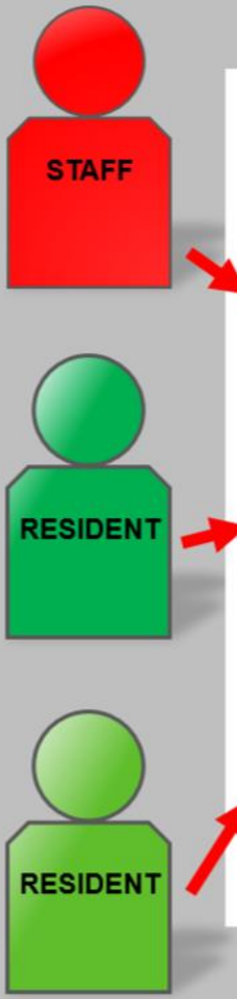

Full Contact Details

The table below shows all contacts the device has had. Please refer to local visitor logs for any visitor devices shown in this report.

Device ID: AC233F66482E | Staff | Trial Number 464696

**Device ID: AC233F664262 | Resident | Trial Number 913372**			
Duration (Minutes)	Start Time	End Time	Room
11	08/10/2020 10:03	08/10/2020 10:14	1st Floor - Canteen behind TV
11	08/10/2020 10:03	08/10/2020 10:14	1st Floor - Canteen behind TV
11	13/10/2020 09:07	13/10/2020 09:18	2nd Floor - Kitchen behind TV
11	13/10/2020 09:07	13/10/2020 09:18	2nd Floor - Kitchen behind TV

**Device ID: AC233F66431F | Resident | Trial Number 923638**			
Duration (Minutes)	Start Time	End Time	Room
13	10/10/2020 11:03	10/10/2020 11:16	1st Floor - Canteen behind TV
13	10/10/2020 11:03	10/10/2020 11:16	1st Floor - Canteen behind TV
